# *Ligustrum lucidum* Leaf Extract-Assisted Green Synthesis of Silver Nanoparticles and Nano-Adsorbents Having Potential in Ultrasound-Assisted Adsorptive Removal of Methylene Blue Dye from Wastewater and Antimicrobial Activity

**DOI:** 10.3390/ma15051637

**Published:** 2022-02-22

**Authors:** Mujaddad Sultan, Maria Siddique, Romana Khan, Ahmed M. Fallatah, Nighat Fatima, Irum Shahzadi, Ummara Waheed, Muhammad Bilal, Asmat Ali, Arshad Mehmood Abbasi

**Affiliations:** 1 Department of Environmental Sciences, COMSATS University Islamabad, Abbottabad Campus, Abbottabad 22060, Pakistan; mujaddad0004@gmail.com (M.S.); romanakhan@cuiatd.edu.pk (R.K.); mbilal@cuiatd.edu.pk (M.B.); 2 Department of Chemistry, College of Science, Taif University, P.O. Box 11099, Taif 21944, Saudi Arabia; a.fallatah@tu.edu.sa; 3 Department of Pharmacy, COMSATS University Islamabad, Abbottabad Campus, Abbottabad 22060, Pakistan; nighatfatima@cuiatd.edu.pk; 4 Department of Biotechnology, COMSATS University Islamabad, Abbottabad Campus, Abbottabad 22060, Pakistan; irumayaz@cuiatd.edu.pk; 5 Institute of Plant Breeding and Biotechnology, Muhammad Nawaz Shareef University of Agriculture, Multan 60000, Pakistan; ummara.waheed@mnsuam.edu.pk; 6 Department of Environmental Sciences and Engineering, School of Environmental Studies, China University of Geosciences, Wuhan 430078, China; asmat@cug.edu.cn

**Keywords:** methylene blue, *Ligustrum lucidum*, silver nanoparticles, nanocomposites, adsorption, wastewater, antimicrobial

## Abstract

Present study was conducted to investigate the adsorption and ultrasound-assisted adsorption potential of silver nanoparticles (AgNPs) and silver nanoparticles loaded on chitosan (AgCS composite) as nano-adsorbents for methylene blue (MB) removal. AgNPs were synthesized using leaf extract of *Ligustrum lucidum*, which were incorporated on the chitosan’s surface for modification. UV–Vis Spectroscopy, FTIR, XRD, SEM, and EDX techniques were used to confirm the synthesis and characterization of nanomaterials. Batch adsorption and sono-adsorption experiments for the removal of MB were executed under optimal conditions; for fitting the experimental equilibrium data, Langmuir and Freundlich’s isotherm models were adopted. In addition, the antimicrobial potential of the AgNPs and AgCS were examined against selected bacterial and fungal strains. UV–Vis spectroscopy confirmed AgNPs synthesis from the leaf extract of *L. lucidum* used as a reducer, which was spherical as exposed in the SEM analysis. The FTIR spectrum illustrated phytochemicals in the leaf extract of *L. lucidum* functioning as stabilizing agents around AgNPs and AgCS. Whereas, corresponding crystalline peaks of nanomaterial, including a signal peak at 3 keV indicating the presence of silver, were confirmed by XRD and EDX. The Langmuir model was chosen as an efficient model for adsorption and sono-adsorption, which exposed that under optimum conditions (pH = 6, dye initial concentration = 5 mg L^−1^, adsorbents dosage = 0.005 g, time = 120 min, US power 80 W), MB removal efficiency of AgNPs was >70%, using ultrasound-assisted adsorption compared to the non-sonicated adsorption. Furthermore, AgNPs exhibited promising antibacterial potential against *Staphylococcus aureus* with the maximum zone of inhibition (14.67 ± 0.47 mm). It was concluded that the green synthesis approach for the large-scale production of metallic nanoparticles is quite effective and can be recommended for efficient and cost-effective way to eradicate dyes, particularly from textile wastewater.

## 1. Introduction

Synthetic dyes are extensively used in different industries, including textiles, leather, food, paper, and pulp. They are classified into various categories, such as acidic, basic, non-ionic, reactive, dispersed, and cationic-basic dyes. Approximately 700,000 tons of 10,000 different types of dyestuffs are produced annually, and it is estimated that 150 tons of these dyes are released into the aquatic environment worldwide [1]. Reactive dyes are colored organic substances, which are cheap, have strong holding properties, and are extensively used specifically in the textile industries in the dyeing of cellulose like cotton and wool [2]. These dyes are non-biodegradable, persistent due to the fact of their synthetic origin and complex chemical properties and structures [2,3]. Discharge of these dyes into water bodies is deleterious to aquatic biodiversity [4,5], and they also possess negative effects on humans health [6,7,8,9,10]. Therefore, it is imperative to provide effective methods for removal of such toxins from the environment [10]. Though many conventional techniques have been employed to remove the harmful toxins from the effluents like reverse osmosis, ion exchange, micro- and ultra-filtration, oxidation, solvent extraction, and electrodialysis, but these methods are exorbitant and tedious [11,12], whereas treatment of colored compounds through adsorption was found to be most effective [13,14], as it has emerged as a promising and versatile water purification technique that can balance high pollutant removal efficiency and the possibility to treat large quantities of water as reported elsewhere [15,16,17]. Moreover, it is an excellent method because of its simple operation, easy use, immense pollution holding capacity, extensive scalability of adsorbents, and high-performance efficiency operations. Adsorption efficiency is highly dependent on the selection of adsorbents that should be cost-effective, readily available, has high reuse potential and low toxicity, and is eco-friendly [16,17,18]. Carbon-based adsorbents, due to the presence of various functional groups for the effective and safe removal of toxins from polluted water, are immensely demanded materials [19]. In addition to other adsorbents, chitosan (an innocuous biopolymer) is the second most abundantly available natural polysaccharide that possesses excellent nontoxic and antimicrobial properties [20]. It has been widely utilized in adsorption due to the fact of its intrinsic properties [21]. Functional groups of chitosan (i.e., amino (–NH_2_) and hydroxyl (–OH)) are mainly responsible for its reactivity as an excellent natural adsorbent with an overall adsorptive capacity [22]. Despite the flexibility of the adsorption behavior, it is unstable in acidic solutions and forms a gel [23]. Hence, it is imperative to develop methods for its stabilization during application. Modification of the chitosan’s surface through nanomaterials can ameliorate the stability of polymeric material [24]. A nanomaterial is a nanosized material with an extremely large surface area, improved reactivity, or high porosity with good mechanical properties. Additionally, another important aspect of chitosan is being used as supporting material, as it acts as a stabilizing matrix to embed the as-synthesized powdery metallic nanoparticles [25]. A combination of the abovementioned advantages through the application of ultrasound further increases mass transfer by raising the diffusion coefficient by best dispersion of adsorbent and probability through opening the porosity of the adsorbent leads to a notable increase in the efficacy of the adsorption procedure [26]. Notably, ultrasound waves can accelerate chemical processes due to the acoustic cavitational phenomenon manifested by transverse propagation of pressure waves along with the liquid [27,28]. Ultrasound waves and their secondary effect enhance the mass transfer through the convection pathway between the active sites of the adsorbate, without a notable change in the equilibrium characteristics of the adsorption/desorption system [26]. Thus, the ultrasonic waves can also increase the kinetics of adsorption.

In the era of modern nanoscience and nanotechnology, metal nanocomposite synthesis is a promising research area. Moreover, because of their apparent physicochemical properties, metal nanocomposites have been utilized in several applications such as adsorption [29]. Among metallic nanoparticles, silver nanoparticles (AgNPs) are unique due to the fact of their exceptional properties, i.e., good electrical conductivity, photoelectrochemical activity, and strong reduction power. Various methods are used to synthesize AgNPs including ultrasonic radiation [30], chemical vapor deposition [31], laser ablation [32], evaporative cooling [33], impregnation [34], co-precipitation [35] deposition–precipitation [36], microwave [37] and sol-gel [38]. But there has been the need to synthesize nanoparticles in a more inexpensive and eco-friendly way, as physical methods involve high energy consumption and considerable capital cost [39,40,41]. The chemical synthesis of AgNPs is non-environmentally friendly, as it requires toxic solvents and stabilizing and reducing agents [29,42]. Meanwhile, the green synthesis process utilizes natural products viz. enzymes, plants, fungi, and algae as reductants and stabilizers to synthesize AgNPs [42,43]. Various plants have been investigated for the synthesis of silver metal (Ag_0_) nanoparticles from silver ions (Ag+). For instance, in recent studies, flowers, fruits, and leaf extract of different plants such as *Gmelina arborea* [44], *Allium cepa* [45], *Thymbra spicata* [46], *Convolvulus arvensis* [47], *Piper chaba* [48], and *Phaseolus vulgaris* [43] have been used to synthesize AgNPs and their application in pollutants removal. These AgNPs can be used as an effective agent for removing methylene blue dye elsewhere [49], Moreover some silver based nanocomposites, i.e., poly(acrylic acid), reduced graphene oxide, activated carbon, and chitosan have been employed in adsorptive removal of MG dye [50] but the ultrasound-assisted adsorption potential of these nanomaterials has not previously been explored, particularly for the removal of MB dye. Given its widespread use [51,52], the present study aimed at the synthesis of AgNPs and AgCS for MB removal.

*Ligustrum lucidum* W.T. Aiton, also known as Chinese Privet, is a flowering plant that belongs to the olive family (*Oleaceae*). Various bioactive compounds such as polyphenolics, triterpenes, secoiridoid glucosides, and volatile elements have been reported in *L. lucidum* fruits that have various pharmacological effects [53]. The synthesis of AgNPs using fruit extract of *L. ovalifolium* and their evaluation for cytotoxic effects against ovarian carcinoma cells has been reported [53]. But to the best of our knowledge, the synthesis of AgNPs and AgNP-loaded chitosan (AgCS nanocomposite) using leaf extract of *L. lucidum* has rarely been investigated so far. Therefore, the present study was intended with the aim (a) to synthesize AgNPs and AgCS using leaf extract of *L. lucidum* and (b) to explore their ability as remediation agents (bio-adsorbent and antimicrobial) for the removal of MB dyes from polluted water.

## 2. Materials and Methods

### 2.1. Materials

Silver nitrate (AgNO_3_), methylene blue (MB), chitosan (CS), and methanol of analytical grade were purchased from Merck, Pakistan. All glassware was washed with aqua regia and rinsed with deionized (DI) water. All aqueous solutions were prepared using double distilled water.

### 2.2. Plant Collection and Drying

**Figure 1:**
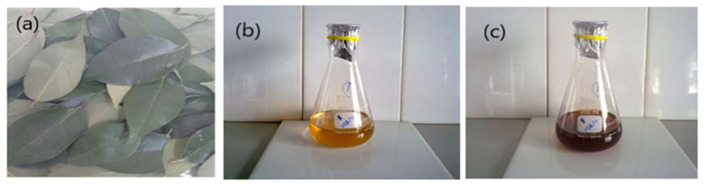
(**a**) *L. Lucidum* plant leaves; (**b**) Leaf extract and aqueous solution of AgNO_3_; (**c**) synthesis of silver nanoparticles.

Fresh leaves of *L. lucidum* (Figure 1a), were collected from COMSATS University Islamabad, Abbottabad Campus, Pakistan. Leaves were thoroughly rinsed with distilled water, shade-dried at room temperature, and converted into a fine powder using an electric grinder.

### 2.3. Ultrasound-Assisted Extraction (UAE) of Leaf Extract

A dried powdered sample of leaves (25 g) was mixed with 300 mL methanol (80%). The ultrasound-assisted extraction (UAE) was carried out in a sonication water bath for 60 min at 25 ± 5 °C at an operating frequency of 40 kHz (100 W). The extract was filtered with Whatman filter paper No. 5 and concentrated using a rotary evaporator at 28 °C for 3 h [54]. The semisolid crude extract was aged for a few days at room temperature to evaporate moisture [55].

### 2.4. Green Synthesis of AgNPs and AgCS Composites

A lab-scale biosynthetic approach was adopted for the AgNPs and AgCS synthesis. Briefly, an aqueous stock solution of AgNO_3_ (1 M, 10 mL) was prepared in distilled water and properly covered with aluminum foil to prevent the auto-oxidation of silver. Different concentrations of crude plant extract (50, 250, and 500 mg), was mixed in 50 mL methanol. Freshly prepared dilutions of AgNO_3_ solution. (1, 5, and 10 mM) were mixed with different concentrations of the leaf extract to synthesize silver nanoparticles as follows—1:1 solution: 1 mM AgNO_3_ (50 mL) solution was mixed in 50 mg methanolic extract (50 mL); for the 1:5 solution, 1 mM solution was mixed in 250 mg methanolic extract; similarly, for the 1:10 solution, 1 mM solution was mixed in 500 mg methanolic extract. Similarly, the 5:1, 5:5, 5:10, 10:1, 10:5, and 10:10 solutions were prepared (Table 1). The mixture of extract and AgNO_3_ was kept in dark for 24 h in an incubator at 37 °C until a change in the color of the mixture indicated the presence of silver nanoparticles. Afterward, the silver nanoparticles solution was centrifuged at 25 °C for 15 min and a 10,000-rpm speed. The supernatant was discarded, and the pellets of nanoparticles were washed with deionized water thrice and freeze-dried for further use [55]. A similar procedure was adopted for the synthesis of AgCS nanocomposites particles. Shortly, 1 g of chitosan was mixed with 0.5 mL of acetic acid in 50 mL of deionized water and was used with a selected concentration of silver nitrate and plant extract to synthesize AgCS nanocomposites.

### 2.5. Characterization

AgNPs and AgCS were characterized to establish their morphology, structure, and surface function [55]. An optical study of synthesized AgNPs and AgCS in colloidal solution was carried out using a T80 + UV/Vis. spectrometer at 200–800 nm. An X-ray diffractogram was obtained from Rigaku, Geiger flex with Cu Kα1 radiation for 2 h values, and the scanning angle was (0–70°). The possible functional groups of the plant extract on the surfaces of the AgNPs and AgCS were identified using FTIR Nicolet iS5 spectrophotometer within a 400–4000 cm^−1^ spectral range, beam splitter Ge coated on KBr, detector DTGS, and resolution 0.8 cm^−1^. The shape and size of nanoparticles and composites were studied using scanning electron microscopy (SEM), Jeol Japan JSM-IT 100 (Tokyo, Japan), while the elemental composition was examined by energy-dispersive spectroscopy (EDX).

### 2.6. Dye Degradation Studies

A stock (500 mg L^−1^) solution of MB dye was used to prepare standards of 5, 10, 25, and 50 mg L^−1^ and the pH was adjusted using diluted HCl and NaOH (0.1 M), respectively. The adsorption and ultrasound-assisted adsorption ability of the synthesized AgNPs and AgCS was accessed by their efficiency to remove MB dye. To attain the adsorption–desorption equilibrium, a known concentration of MB (10 mg L^−1^) in a 50 mL working solution and a definite quantity of adsorbents (0.005 g) were agitated for in a conical flask for 3 h.

The ultrasound-assisted adsorption was carried out in a digital ultrasonic bath for 120 min, at 30–35 °C, 80 W power, and a 40 kHz frequency. The effect of operative parameters for adsorption and ultrasound-assisted adsorption experiments were contact time (0–120 min), varying pH (4, 6, 8, and 10), different dye concentrations (5, 10, 25, and 50 mg L^−1^), temperature (30–35 °C), and ultrasonic power (80 W) at 40 kHz frequency were evaluated and optimized over a fixed adsorbent dose (0.005 g). At an interval of 30 min, absorbance was checked for each solution at a specific wavelength of ƛ_max_ 661 nm using a UV–Vis spectrophotometer. Dye removal efficiency (R) was estimated for adsorption and sono-adsorption using the following equation:

R (%) = C_i_ − C_f_/C_i_ ∗ 100 (1)

where C_i_ = initial dye concentration before adsorption, and C_f_ = final dye concentration after adsorption and sono-adsorption process.

### 2.7. Isotherm Analysis

Langmuir isotherm explained the interaction between contaminant and adsorbent, the adsorption capacity of adsorbent, monolayer, or multilayer formation of adsorbate molecules on homogeneous or heterogeneous distribution of active adsorption sites with similar or dissimilar energy levels at a constant temperature [56,57]. Adsorption and sono-assisted adsorption of MB dye onto silver nanoparticles isotherms attained for dye concentrations (5, 10, 25, and 50 mg L^−1^) were used to fit the linear models as described in Equation (2):

C_e_/q_e_ = 1/K_L_q_ecal_ + C_e_/q_ecal_ (2)

R_L_ = 1/1 + K_L_C_0_ (3)

where C_e_ is the residual concentration of MB dye and q_e_ is the adsorption capacity of silver nanoparticles at equilibrium. K_L_ reveals the Langmuir constant, and q_ecal_ represents adsorption capacity predicted by the Langmuir isotherm. C_0_ indicates the initial concentration of dye. Equation (3) unfolds the favorable (0 < R_L_ > 1), unfavorable (R_L_ > 1), irreversible (R_L_ = 0), and linear (R_L_ = 1) adsorption [58]. The linear isotherm of the Freundlich model [57] is described in Equation (4):

log (q_e_) = log (k_f_) + 1/n log (C_e_) (4)

where k_f_ indicates the Freundlich constant, and n determines the bonding energy between dye molecule and nanoparticles.

### 2.8. Assessment of the Antimicrobial Activity of AgNPs and AgCS

The agar well diffusion method, as explained in literature [55], was adopted to determine antimicrobial activities of AgNPs, AgCS, and control (chitosan). The antibacterial assay was analyzed on two bacterial strains i.e., *Staphylococcus aureus* ATCC# 6538 (and Methicillin-resistant *S. aureus* (MRSA) and used to test antibacterial potential, and *Candida albicans* ATCC #9002 strain was used in the antifungal activity of AgNPs and AgCS.

The inoculum was prepared from 24 h old culture of selected bacteria in nutrient broth, and turbidity of the bacterial cultures were analyzed and compared with Mac Farland’s turbidity standard of 0.5 (10^6^ colony-forming unit (CFU) per mL). The nutrient agar (Merck, Darmstadt, Germany) was prepared according to the manufacturer’s instructions and a standardized suspension of bacteria was used for nutrient agar plates. Petri plates with bacterial culture were used and wells of 6 mm were made with a sterile cork borer and sealed. The AgNPs, AgCS, and chitosan were mixed in dimethyl sulfoxide (DMSO) (Sigma-Aldrich, Burlington, MA, USA) at a concentration rate of 3 mg/mL. Ciprofloxacin (0.5 mg/mL) was used as a standard antibiotic (positive control), and pure DMSO was used as a negative control. Each well contained 20 µL of each: a sample (60 µg), positive control (15 µg), and negative control. These plates were incubated at 37 °C, for 24 h and zones of inhibition (ZOI mm) were measured. Triplicate analyses were conducted for each sample.

## 3. Results and Discussion

### 3.1. Formation of AgNPs and AgCS

Initially, the mixture of the leaf extract and aqueous solution of AgNO_3_ was appeared to be light orange in color (Figure 1b). This mixture was kept in dark for 24 h at normal temperature and change in color from brown to dark brown was observed (Figure 1c). This change in the color of the mixture was considered an indication of AgNPs synthesis. Similarly, the color change was also noticed during the formation of Ag-loaded chitosan (AgCS) composites, i.e., particles, and this color change might be due to the excitation of the surface plasmon vibrations/resonance in AgNPs. In addition, the reduction of silver ions into silver atoms by various phytochemicals viz. saponins, flavonoids, and alkaloids in plant extract during synthesis of AgNPs is also indicated by a change in color [43,59].

### 3.2. Characterization of Synthesized AgNPs and AgCS

#### 3.2.1. Ultraviolet–Visible Analysis

**Figure 2:**
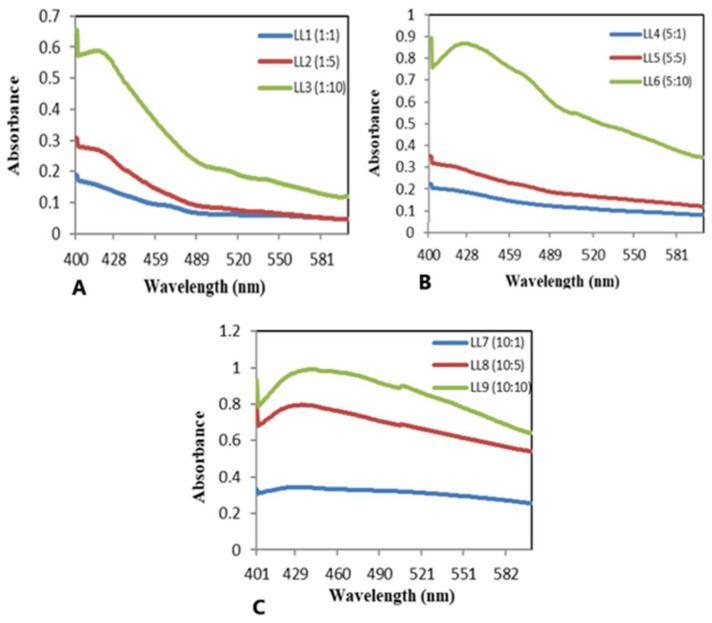
UV–Vis spectra of AgNP with different concentrations of silver nitrate and plant extract: (**A**) *Ligustrum lucidum* (LL) silver nanoparticles prepared with 1 mM AgNO_3_ and 1, 5, and 10 mg mL^−^^1^ of plant extract (LL1, LL2, LL3); (**B**) *Ligustrum lucidum* (LL) silver nanoparticles prepared with 5 mM AgNO_3_ and 1, 5, 10 mg ml^-1^ of plant extract (LL4, LL5, LL6), (**C**) *Ligustrum lucidum* (LL) silver nanoparticles prepared with 10 mM AgNO_3_ and 1, 5, 10 mg ml^-1^ of plant extract (LL7, LL8, LL9).

The UV–Vis spectral analysis was used to validate the synthesis of AgNPs and AgCS. The presence of AgNPs and AgCS was observed by the absorption spectrum of the reaction mixture (0 min and after 24 h) in the wavelength range from 400–450 nm (Figure 2). The spectrum of the solution specified that the surface plasmon resonance derived from the nanoparticles was around 402–428 nm, which is a typical absorption region of silver nanoparticles. The results revealed that AgNO_3_ and plant metabolites play an important role in the synthesis of nanoparticles (NPs) [60]. When a low concentration of AgNO_3_ was applied (1 and 5 mM) the band for AgNPs appeared in the range of 402–420 nm (Figure 2b). Beyond 5 mM, the intensity stabilized due to the unavailability of plant extract to react with Ag^+^. Thus, to obtain optimal quality and quantity of AgNPs, low concentrations of AgNO_3_ were used (Figure 2c). Similarly, an increase in the concentration of plant extracts enhanced AgNPs synthesis. However, excessive amounts of plant extract may form aggregates, and such aggregation of the nanoparticles is highly dependent upon the concentration of biomolecules in the plant extract [61]. These biomolecules act as reducing/stabilizing/capping agents at suitable concentrations and reduce or cap the surfaces of the nanoparticles and protect them against aggregation [62]. The nanoparticles form caps, specifically when the amount of plant extract is less and has a low concentration of biomolecules involved in nanoparticles synthesis. Our study exposed that: 5:10 is an optimal ratio between leaves extract and silver nitrate solution (Figure 2b). AgNPs prepared at this ratio were used for characterization, dye adsorption, and antimicrobial studies.

It has been documented that AgCS formation increases while increasing the concentration of AgNO_3_, but shows an aggregation of NPs on the surface of chitosan [60]. To synthesize AgCS, the silver nitrate ratio was kept fixed at 5 mM using different concentrations of plant extract (1, 5, 10 mg mL^−^^1^) and 1 g of chitosan. UV–Vis spectral analysis of synthesized AgCS, revealed that bands appeared in the range of 422–483 nm for AgCS, and this band shifted from lower to higher wavelengths as shown in Figure 2c.

#### 3.2.2. Fourier Transform Infrared (FTIR) Analysis

**Figure 3:**
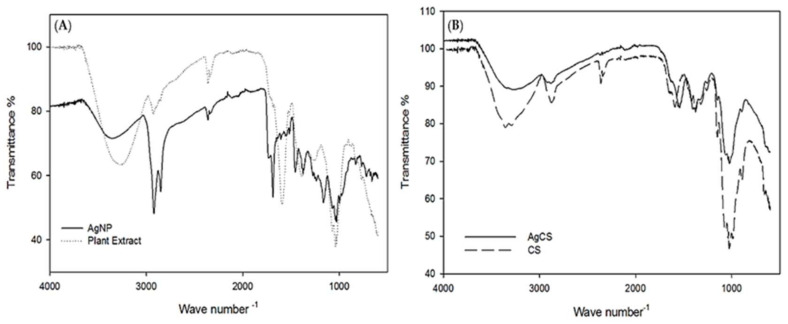
FTIR analysis of (**A**) synthesized AgNPs and plant extract of *L. lucidum* (**B**) of pure CS and AgCS.

The FTIR spectra of the *L. lucidum* leaf extract and AgNPs (Figure 3a) depicted a similar spectrum. Both had bands at 3385.1 and 3293.6, cm^−1^ that corresponded to O–H stretching, [63] those at 2920.6, 2913.2, 2855.54, and 2847.8 cm^−1^ corresponded to C–H stretching, [64] those at 1462.5, 1382.6, 1375.3, 1171.4, and 1025.23 cm^−1^ were consistent with C–H bending vibration, C–O functional groups at 1681.34 and 1601.2 cm^−1^ conformed to C=O and aromatic C=C double-bond functional groups, and those at 711.4, 602.2, and 595.2 cm^−1^ [65,66] corresponded to the deformation vibration of C–H bonds in the phenolic rings. These results revealed that the plant extract contains several functional groups such as –OH, –CH, C=O, and C=C, which are important for the reduction and stabilization of the biosynthesized AgNPs [67].

The main peaks of pure chitosan in (Figure 3b) can be designated as: 3358.1 (O–H and N–H stretching vibrations), 2870.2 (C–H vibration), 1641 (N–H deformation vibration), 1594.4 (stretching vibration of an amino group of chitosan), 1397 (C–H symmetric bending vibration), and 1039.7 cm^−1^ (C–O stretching vibration). The IR spectrum of the AgCS nanocomposites exhibited similar functional groups to those of pure chitosan. However, in the AgCS, the width of the OH stretching band at 3358.1 cm^−1^ and the intensity of the C–O stretching band at 1039.7 cm^−1^ decreased compared to pure CS. There was a slight shift in the stretching vibration of the N–H deformation vibration and C–O stretching vibration, which may be due to the interaction of silver nanoparticles with CS.

#### 3.2.3. X-ray Diffraction Analysis (XRD)

**Figure 4:**
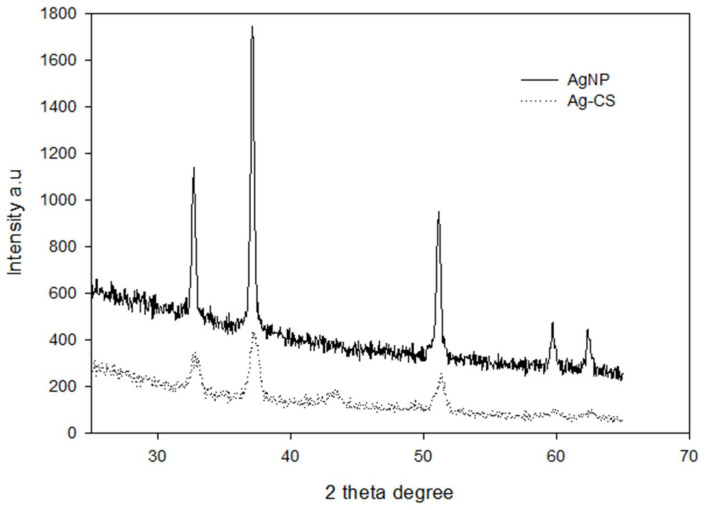
XRD pattern of biosynthesized AgNPs and AgCS.

The crystallinity of the AgNPs and AgCS was determined by XRD analyses (Figure 4) following the procedure as explained previously in the literature [67]. The X-ray diffractogram illustrated five prominent peaks at 2θ values of 32.71°, 37.18°, 53.18°, 59.71°, and 64.34° corresponding to the (122), (111), (142), (241), and (220) Bragg’s reflections assigned to lattice planes of face-centered cubic (FCC) structure of metallic silver as reported in the literature [68]. These results indicate that Ag^+^ was reduced to Ag^0^ and resulted in pure crystallized metallic silver. In contrast to the diffraction intensity conforming elemental silver in the XRD pattern of AgCS, no significant change for the diffraction pattern of AgCS was found but for the decreased intensity. This decrease in characteristic peak intensity of AgCS in the XRD patterns may be because compared to pure silver, as the silver quantity was reduced in AgCS composite.

#### 3.2.4. Scanning Electron Microscopy and EDX

**Figure 5:**
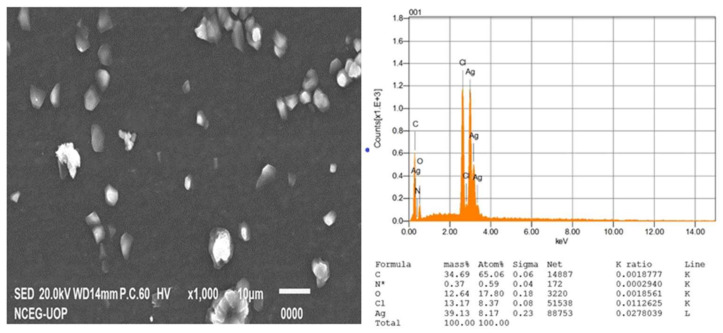
(**a**) SEM images and (**b**) EDX spectra of AgNPs prepared from *L. lucidum*.

**Figure 6:**
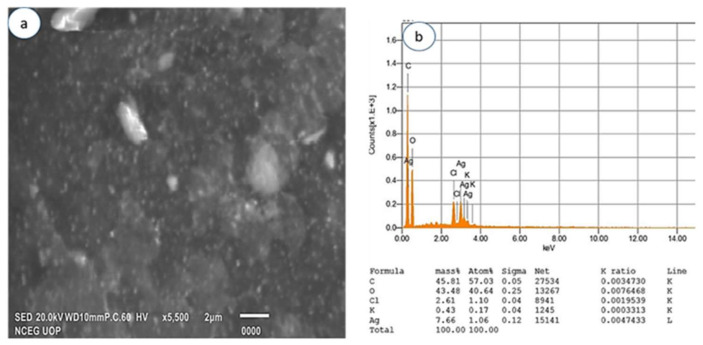
(**a**) SEM images and (**b**) EDX spectra of AgCS prepared from *L. lucidum*.

The SEM analysis was performed to ascertain the morphology of AgNPs and AgCS. In this study, the SEM analysis showed the AgNPs with a spherical morphology (Figure 5a), compared to the morphology of AgCS (Figure 6a), where AgNPs aggregated on the surface of chitosan. EDX analysis gives the qualitative status of elements constituting nanoparticles. In that regard, the elemental profile of synthesized AgNPs exhibited a signal peak at 3 keV as a result of silver (Figure 5b), which is typical for the absorption of silver nanoparticles [67]. The elemental profile also demonstrated the presence of oxygen (12.64%), nitrogen (0.37%), carbon (34.69%), and chlorine (13.17%), while the elemental profile of AgCS was lower at 3 keV compared to AgNPs (Figure 6b).

### 3.3. Adsorptive and Ultrasound-Assisted Adsorptive Performance of AgNPs and AgCS

The synthesized AgNPs and AgCS (with/without chitosan) were applied to remove MB using adsorption and sono-adsorption processes. The experiments were performed in triplicates and the average values of the estimated parameters were reported. The results of the performance tests under different conditions of variable operating parameters, i.e., pH, initial dye concentration, and contact time at fixed concentrations of adsorbents (0.005 g), and the US power (80 W) for AgNPs and AgCS are presented in subsequent sections.

#### 3.3.1. Effect of pH

**Figure 7:**
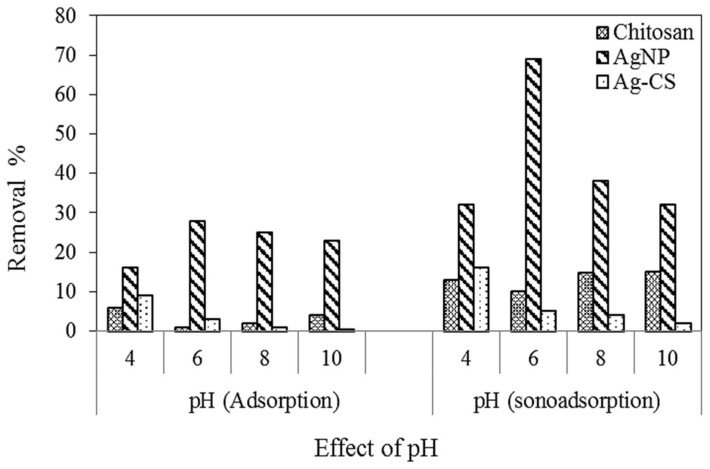
Effect of pH on adsorption and sono-adsorption of MB using chitosan, AgNPs, and AgCS (MB concentration = 5 mg L^−^^1^, chitosan concentration = 0.005 g, AgNPs dosage = 0.005 g, AgCS = 0.005 g, time = 120 min, US power 80 W).

The pH of the solution plays a vital role in the efficiency of the adsorption processes. The effect of pH was investigated by varying the pH range (4, 6, 8, and 10) for removing MB onto chitosan, AgNPs, and AgCS using adsorption and sono-adsorption processes. The experimental results for MB uptake for initial pH values in the range 4 to 10 are shown in Figure 7, which exposed that at 4–6 pH values, high retention occurs compared to the others due to the electrostatic attraction between adsorbate and adsorbent in the adsorption process. [69]. In the present study, for pH values ranging from 4 to 6, a slight increase in MB removal was observed. Therefore, optimum pH of 6 was selected. If we compare different adsorbents, their efficiency was in the following order: AgNPs > Chitosan > AgCS. The possible explanation is taking the same amount of adsorbent, AgNPs have more adsorption sites due to the small size and surface area compared to chitosan and AgCS composite. In composite, the Ag active sites were not available due to their adsorption on Chitosan. Furthermore, compared to adsorption, sono-adsorption efficiency was found to be better for all adsorbents, because ultrasonic waves reduce the agglomeration of nanoparticles.

#### 3.3.2. Effect of Contact Time

**Figure 8:**
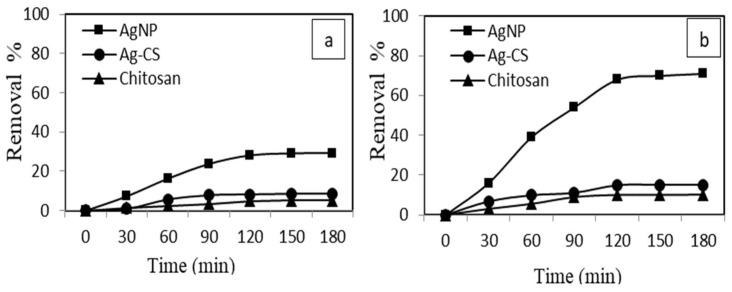
Effect of contact time on (**a**) adsorption and (**b**) sono-adsorption of (MB) using chitosan, AgNPs and AgCS (MB concentration = 5 mg L^−^^1^, Adsorbent’s concentration = 0.005 g, pH = 6, min, US power 80 W).

The equilibrium time of chitosan, AgNPs, and AgCS nano adsorbents was determined using adsorption and sono-adsorption processes. As illustrated in Figure 8, MB uptake by AgNPs was increased rapidly during the first hour compared to chitosan and AgCS composite. Because, initially, the surface of AgNPs had many binding sites that enhanced the adsorption of MB. Within 120 min, AgNPs removed 70% and 28% MB using sono-adsorption and adsorption processes, respectively Figure 8a, b. We observed that the adsorption process was hindered after 120 min and was almost stable after 150 min. This change in the rate of the adsorption process may be due to (a) saturation of adsorption sites and (b) a steady decrease in the concentration gradient between AgNPs and bulk solution. This trend was similar to that reported in the literature for dye adsorption from aqueous solution [60,70]. However, in the sono-adsorption process, ultrasonic irradiation might increase the mass transfer rate of solute around the solid–liquid interfaces because of the high-pressure shock waves in the violent collapse of cavitation bubbles [71]. Thus, sonication develops equilibrium in a short time than the conventional operation.

#### 3.3.3. Effect of Initial Dye Concentration

**Figure 9:**
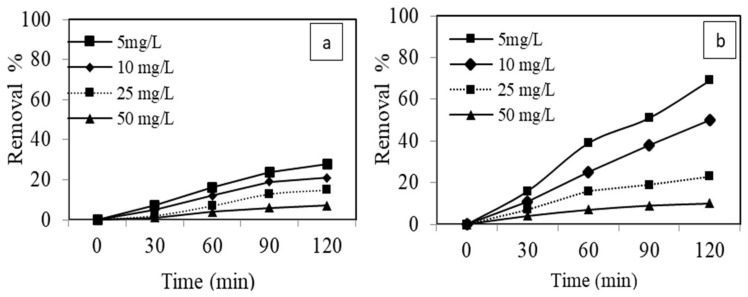
Effect of initial dye concentration on (**a**) adsorption and (**b**) sono-adsorption of (MB) using AgNPs.(AgNPs dosage = 0.005 g, pH = 6, US power = 80 W, and time = 120 min).

The adsorption process is greatly influenced by the initial concentration of adsorbate. Herein, the effect of initial concentration was explored at variable concentrations of dye i.e., 5–50 mg L^−1^ at optimum adsorbent dosage, pH, and contact time on adsorption and sono-adsorption processes. The removal efficiency of dye on AgNPs as shown in Figure 9, decreased as the adsorbate initial concentration increased from 5 to 50 mg L^−1^, and maximum adsorption was observed in the sono-adsorption processes as sonication leads to more solute mass transfer via acoustic cavitation’s [70]. Because, at a lower concentration, nearly all dye molecules interact with available binding sites of the adsorbent. At a higher initial concentration of dye, the binding sites became saturated and adsorption capacity shows no improvements. Moreover, when the volume and mass of adsorbent solution were kept constant, the ratio of active sites to the dye concentration became lower and resulting in a decrease in the percentage of dye removal [71].

### 3.4. Isotherms

The isotherms model parameters are listed in Table 1. The coefficient of determination (*R*^2^) reveals the goodness of fit of experimental data on both models. Based on *R*^2^, Langmuir isotherm better describes the MB dye removal during both adsorption and sono-adsorption processes compared to Freundlich isotherm, revealing monolayer adsorption on homogeneous active sites for MB dye adsorption on the surface of nanoparticles [72]. In the sono-adsorption system, more adsorption capacity and improved MB dye adsorption data fitness to the Langmuir isotherm indicate that ultrasound irradiation increases the homogeneous distribution of active sites for better uptake of MB dye molecules [73]. This enhanced homogeneous distribution of active sites can be demonstrated in terms of more accurate fitting of adsorption data in sono-adsorption with *R*^2^ value of 0.9926 (Table 2). The favorability of silver nanoparticle adsorbent can be perceived with R_L_ (zero to unity) [74].

### 3.5. Antimicrobial Performance Assessment of AgNPs and AgCS

The AgNPs (M2) of the *L. lucidum* plant showed significant activity against bacterial strain: *S. aureus* with the maximum zone of inhibition (21.00 ± 0.82 mm). Correspondingly, AgCS (M3), was also active against *S. aureus* with the zone of inhibition (14.67 ± 0.47 mm). However, M2 and M3 were moderately active for MRSA in the antibacterial assay. Furthermore, AgNPs and AgCS depicted significant potential against *Candida* strains with a maximum zone of inhibition (8.00 ± 0.82 and 9.00 ± 0.82 mm, respectively) as mentioned in Table 3. The chitosan (M1) sample was inactive for both antibacterial and antifungal assays, as reported elsewhere [75].

The findings of the antibacterial assay revealed that there was a clear increase in the activities of AgNPs against *S. aureus*. These findings were analogous to the previous reports in the literature that silver nanoparticles had strong antibacterial activities [55,76,77]. Composite was also active for *S. aureus*, while unaccompanied nanoparticles of *L. lucidum* were more significantly active. These results confirm the findings of researchers that nanoparticles are more efficient against pathogens than composite [78]. However, both AgNPs and composite of *L. lucidum* were moderately active against *C. albicans* strain with the improved activity of composite [79,80]. It was found that the AgNPs synthesized at 5 mM concentration displayed strong activities. This result endorses the fact that the quantity of metal concentration and plant extract in reaction influence the process of biosynthesis, which may change the sizes, shapes, and activity of metallic nanoparticles [81].

The antimicrobial action of silver nanoparticles is based on various mechanisms. Such as the surface binding of the bacterial cell wall and membrane penetration into the cytoplasm. This causes disruption of organelles and ultimately production of free radicals (ROS and RNS) in the cell that damage DNA and lead to cell death [82]. The interaction of chitosan and composite is based on mechanisms of electrostatic interaction of charges on chitosan and microorganisms. Chitosan molecular weight controls its penetration into the nuclei of microorganisms and binding with DNA. This causes suspension of protein synthesis and leads to cell death [83].

## 4. Conclusions

We established a simple, low-cost, eco-friendly, and green route to synthesize AgNPs and AgCS for the first-time using *L. lucidum* leaf extract. The composition and characteristics of nanoparticles and composite were confirmed by SEM, FTIR, EDX, and XRD techniques. Generally, the removal efficiency of AgNPs and AgCS increased within an increase in time, while decreasing with an increase in the concentration of dye and pH of the solution. However, AgNPs were found to be efficient compared to AgCS and chitosan. Ultrasonically driven adsorption process showed 70% MB dye removal using AgNPs at optimum conditions. The Langmuir model was chosen as an efficient model for adsorption and sono-adsorption of MB dye. Moreover, the AgNPs proved to possess good antimicrobial potential. Our findings suggest that the green synthesis of nanomaterials from *L. lucidum* leaves can be used as a potential material in dye adsorption and as an effective antibacterial agent for water treatment.

## Figures and Tables

**Table 1:** Code assigned to different concentrations of silver nitrate and Ligustrum lucidum leaf extract.

Sample Number	Code	AgNO_3_ (mM) in 50 mL	Extract Concentration (mg/mL) in 50 mL
1	LL1	1	1
2	LL2	1	5
3	LL3	1	10
4	LL4	5	1
5	LL5	5	5
6	LL6	5	10
7	LL7	10	1
8	LL8	10	5
9	LL9	10	10

**Table 2:** Constants and parameters for isotherms fitness.

Isotherm Models	Parameters	Adsorption	Sono-Adsorption
Langmuir	q_exp_	40	60
	q_m_	49.26	50.51
	R_L_	0.16	0.01
	K_L_	0.11	2.20
	R^2^	0.9782	0.9926
Freundlich	k_F_	7.65	15.75
	n	2.11	8.83
	R^2^	0.9116	0.5566

**Table 3:** Antibacterial and antifungal activities of chitosan, AgNPs, and AgCS.

Samples	Zone of Inhibition (mm)		
	**Antibacterial**		**Antifungal**
	**MRSA**	**SA**	**Ca**
M1	0.33 ± 0.47	0.33 ± 0.47	0.33 ± 0.47
M2	7.33 ± 0.47	21.00 ± 0.82	8.00 ± 0.82
M3	8.67 ± 1.25	14.67 ± 0.47	9.00 ± 0.82

## Data Availability

Not applicable.
